# Associating transcriptional modules with colon cancer survival through weighted gene co-expression network analysis

**DOI:** 10.1186/s12864-017-3761-z

**Published:** 2017-05-09

**Authors:** Rong Liu, Wei Zhang, Zhao-Qian Liu, Hong-Hao Zhou

**Affiliations:** 1 Department of Clinical Pharmacology, Xiangya Hospital, Central South University, Changsha, 410008 People’s Republic of China; 2 Institute of Clinical Pharmacology, Central South University; Hunan Key Laboratory of Pharmacogenetics, Changsha, 410078 People’s Republic of China

**Keywords:** Colon cancer, Gene expression profiling, Systems biology, WGCNA, Biomarker

## Abstract

**Background:**

Colon cancer (CC) is a heterogeneous disease influenced by complex gene networks. As such, the relationship between networks and CC should be elucidated to obtain further insights into tumour biology.

**Results:**

Weighted gene co-expression network analysis, a powerful technique used to extract co-expressed gene networks from mRNA expressions, was conducted to identify 11 co-regulated modules in a discovery dataset with 461 patients.

A transcriptional module enriched in cell cycle processes was correlated with the recurrence-free survival of the CC patients in the discovery (HR = 0.59; 95% CI = 0.42–0.81) and validation (HR = 0.51; 95% CI = 0.25–1.05) datasets. The prognostic potential of the hub gene Centromere Protein-A (CENPA) was also identified and the upregulation of this gene was associated with good survival. Another cell cycle phase-related gene module was correlated with the survival of the patients with a KRAS mutation CC subtype. The downregulation of several genes, including those found in this co-expression module, such as cyclin-dependent kinase 1 (CDK1), was associated with poor survival.

**Conclusion:**

Network-based approaches may facilitate the discovery of biomarkers for the prognosis of a subset of patients with stage II or III CC, these approaches may also help direct personalised therapies.

**Electronic supplementary material:**

The online version of this article (doi:10.1186/s12864-017-3761-z) contains supplementary material, which is available to authorized users.

## Background

Colon cancer (CC), the third most common cancer worldwide, is one of the major causes of tumour-related death in the United States [1]. CC is a biologically heterogeneous disease characterised by neoplasms defined by discrete pathological properties and consequent clinical results. Tumour, node and metastasis (TNM) staging is a prognostic classification traditionally used in clinical practice to select patients with CC for adjuvant chemotherapy. However, TNM staging fails to accurately predict recurrence in many CC patients who undergo surgical therapy. For instance, approximately 10 to 20% of patients with stage II colorectal cancer and 30 to 40% of patients with stage III colorectal cancer develop recurrence. Thus, prognostic and predictive factors should be developed to provide reliable information as a basis of medical treatment decisions in routine clinical practices.

Our understanding of the extent of CC complexity has been greatly enhanced through comprehensive studies on molecular biomarkers. The molecular classification of CC is based on a few commonly used biomarkers, such as microsatellite instability (MSI), CpG island methylator phenotype (CIMP), chromosomal instability and BRAF and KRAS mutations [2, 3]. In a large population-based study, five CC subtypes are defined on the basis of the combinations of MSI, CIMP, and BRAF and KRAS mutations, and these subtypes are associated with marked differences in survival [4].

Microarray technology has been applied to investigate gene expression profiles (GEPs) in CC; the extraction of transcriptomics-based prognostic signatures has also been extensively studied [5–8]. To the best of our knowledge, five GEP-related tests, namely, Oncotype DX® Colon Cancer (Genomic Health, Inc.), ColoPrint® (Agendia NV), ColonPRS® (Signal Genetics, LLC), OncoDefender-CRC™ (Everist Genomics, Inc.) and GeneFx Colon (Precision Therapeutics, Inc.), have been developed to examine CC. However, multigene assays for clinical practices, such as risk assessment and adjuvant treatment determination, have yet to be designed [5, 9]. Furthermore, the repeatability of CC-related GEP studies is poor possibly because CC is composed of distinct molecular entities that may be developed through numerous functional biological pathways. As a result, several prognostic signatures may correspond to different entities of CC. Therefore, an early unsupervised consensus hierarchical clustering of genome-wide mRNA levels has prompted researchers to classify CC into six distinct molecular subtypes [10].

The accumulation of large numbers of CC mRNA datasets in several databases, such as GEO, provides an opportunity to reanalyse the gene mRNA expression data derived from different platforms and institutes (termed as meta-analysis) and to define the objective classifications of sample subtypes [11]. Integrated information from multiple studies can highly tolerate the heterogeneity associated with CC and the variability caused by microarray techniques; such information also helps increase statistical power as the number of samples is increased. Meta-analyses have been applied to evaluate single genes in studies on CC; for instance, a meta-analysis has been conducted to identify the correlation of the overexpression of the metastasis associated in colon cancer-1 (MACC1) gene with poor disease-free survival [12]. Agostini et al. conducted an integrative systematic approach for the identification of prognostic biomarkers in rectal cancer [13]. Large CC databases, such as Georgetown Database of Cancer, have been developed to evaluate the association of prognostic biomarkers with recurrence and to identify the subgroup of patients who may benefit from adjuvant chemotherapy [14].

Weighted gene co-expression network analysis (WGCNA) has emerged as an effective method of multigene analysis to discover the relationship between networks/genes and phenotypes. In WGCNA, gene modules are established from mRNA expression data by using unsupervised hierarchical clustering; thus, this technique does not depend on a priori defined gene sets or pathways. The basic concept of WGCNA involves a gene co-expression module, which is a cluster of genes that maintains a consistent expression pattern and possibly shares a common biological regulatory role [15]. WGCNA has been successfully applied to identify networks and biomarkers that can be used to screen, diagnose and treat cancer. This technique has also been used to reveal the microRNA and mRNA expression network implicated in prostate cancer [16] and to identify the co-expression networks related to proastrocytic differentiation in glioma [17]. Wirapati et al. [18] and Clarke et al. [19] conducted WGCNA to identify co-expressed gene modules among breast cancer patients on the basis of multiple microarray-based gene expression datasets; Wirapati et al. [18] and Clarke et al. [19] also explored the relationship of these transcriptional modules with clinical variables, such as tumour size and grade, survival outcomes related to breast cancer as a whole and the corresponding molecular subtypes.

In this study, WGCNA was applied to analyse a dataset obtained from a transcriptome comprising 461 patients with stage II or III CC to identify gene modules and biomarkers (hub genes) for the prognosis of CC patients. Furthermore, our findings were confirmed via a validation dataset with 111 CC patients.

## Methods

### Microarray-based mRNA expression datasets

We analysed two microarray datasets in CC. The raw gene expression data were retrieved from the GEO data repository (http://www.ncbi.nlm.nih.gov/geo/) with the accession numbers GSE39582 [10] and GSE17536 [20]. In addition, we have labelled these datasets on the basis of their GEO accession numbers. The discovery dataset GSE39582 was used to generate network; by contrast, GSE17536 was used as an independent validation dataset. Our survival analysis was restricted to the subgroup of the patients with stage II or III CC because the discovery of reliable prognostic biomarkers is used for this subgroup of patients. A large proportion of patients with stage I CC unlikely provide benefits from adjuvant chemotherapy because of their excellent prognosis after surgical treatment is completed. Most of the stage IV patients who are metastatic may die from the disease; therefore, these patients should be analysed independently for progression-free survival. Moreover, patients without the recorded survival time were excluded from our analysis. After filtering was performed, the discovery and validation datasets respectively containing 461 and 111 samples were originally generated using Affymetrix U133 Plus 2.0 chips. Clinicopathological variables (e.g. age, tumour grade), biomarker gene mutation (BRAF and KRAS) and recurrence-free survival (RFS, defined as the time from surgery to the first recurrence and was cut off in 5 years) were gathered for each dataset (Additional file 1: Table S1). The microarray datasets were processed with Robust Multiarray Average algorithms by utilising the ‘affy’ Bioconductor package. We used the ComBat algorithm to adjust the expression data for potential batch effects [21]. Before conducting WGCNA, we filtered the probes without known gene symbols, and the probe-level expression profiles for the datasets were converted to gene-level expressions by using the collapseRows function to merge probes [22].

### Colon cancer molecular subtypes

The CC patients in our study were divided into five molecular subtypes in accordance with the report of Phipps et al. [4] In brief, patients are classified on the basis of the following combinations of tumour biomarkers: type 1 (MSI-high, CIMP-positive, BRAF mutation-positive, KRAS mutation-negative); type 2 (MSI-low, CIMP-positive, BRAF mutation-positive, KRAS mutation-negative); type 3 (MSI-low, CIMP-negative, BRAF mutation-negative, KRAS mutation-positive); type 4 (MSI-low, CIMP-negative, BRAF and KRAS mutation-negative); and type 5 (MSI-high, CIMP-negative, negative for BRAF and KRAS mutations). The information related to the MSI and CIMP status was obtained as described in the original publication of the discovery dataset [10]. In this dataset, 26 samples were classified as type 1, 6 samples were classified as type 2, 108 samples were classified as type 3, 151 samples were classified as type 4 and 8 samples were classified as type 5. Subsequent subtype survival analyses were not performed on subtypes 1, 2 and 5 because of their small sample sizes. The rest of the 162 samples in the discovery dataset and all of the samples in the validation dataset were not included in any subtype because of the lack of information.

### Co-expression module detection

We selected the top 5000 varying genes from the 461 patients in the discovery dataset after their standard deviations were sorted in an ascending order. The WGCNA in this study was restricted to the 3600 most co-expressed genes from these 5000 genes in the dataset (based on k.total, as described below) by using the R ‘wgcna’ package [23].

The co-expression networks of the selected genes were generated using the following steps. An unsupervised co-expression relationship was initially built on the basis of the adjacency matrix of connection strengths by using Pearson’s correlation coefficients for gene pairs. This matrix was increased to β = 4 based on the scale-free topology criterion (Additional file 1: Figure S1). Based on the scale-free topology criterion, the power β was selected to amplify the strong connections between genes and penalise the weaker connections. The Network connectivity (k.total) of the *i*th gene was defined as the sum of its adjacency with all of the other genes to generate networks. The intramodular connectivity (k.in) was calculated as the summation of adjacency performed over all of the genes in a particular network; hub genes were those with a high network connectivity in a particular group. Modules were identified as gene sets with a high topologic overlap [24]. Average linkage hierarchical clustering was conducted on the basis of a topological overlap matrix (TOM)-based dissimilarity measure; in this technique, the hybrid dynamic tree­cutting method was used to cut branches by using a minimum gene module size of 30 and a cut height of 0.95.

The module eigengenes (MEs) were generated as the first principal component after principal component analysis was performed with the expression data for each co-expressed modules in the 461 samples. Module membership assignment (kME) was determined as Pearson’s correlation coefficient between gene expression values and MEs.

The WGCNA algorithm was described in detail by Zhang Bin et al. [24].

### Survival analysis

The ‘survival’ R package (http://cran.r-project.org/web/packages/survival/index.html) was subjected to survival analysis. The hazard ratio (HR) and the corresponding 95% confidence interval (CI) were calculated using a Cox regression model. Kaplan-Meier survival curves were plotted. RFS was used for the survival endpoints. For module associations, each ME was robustly scaled to obtain −1, 0 and +1 for 2.5, 50 and 97.5% quintiles, respectively; using these values, we can compare different modules. The scaled MEs were then treated as continuous variables. For gene associations, each gene expression was treated as a continuous variable. False discovery rate (FDR) method was used to perform multiple testing corrections. The survival-based gene significance (GS) was defined as minus log 10 of Cox regression *p*-values. Furthermore, hub genes were defined as those with a high network connectivity (k.in), which corresponds to the connect strength (co-expressed) of a specific gene with all the other members in a module. The hub genes highly associated with clinical traits and highly connected to the modules were identified through GS and k.in. In particular, hub genes were obtained on the basis of the following criteria: (i) the value of the k.in is in the top 10 of all of the genes in the module and (ii) GS is greater than 2.

### Functional annotation modules

The overrepresentation in gene ontology (GO) categories was searched to extract further biological insights into the genes belonging to the modules associated with the survival of CC patients. DAVID (http://david.abcc.ncifcrf.gov/) [25] was employed to evaluate the modules for the enrichment of the genes with particular GO biological processes compared with the background list of the human genes and to calculate the enrichment scores of the GO biological process terms.

## Results

### Detection of gene co-expression modules

**Fig. 1:**
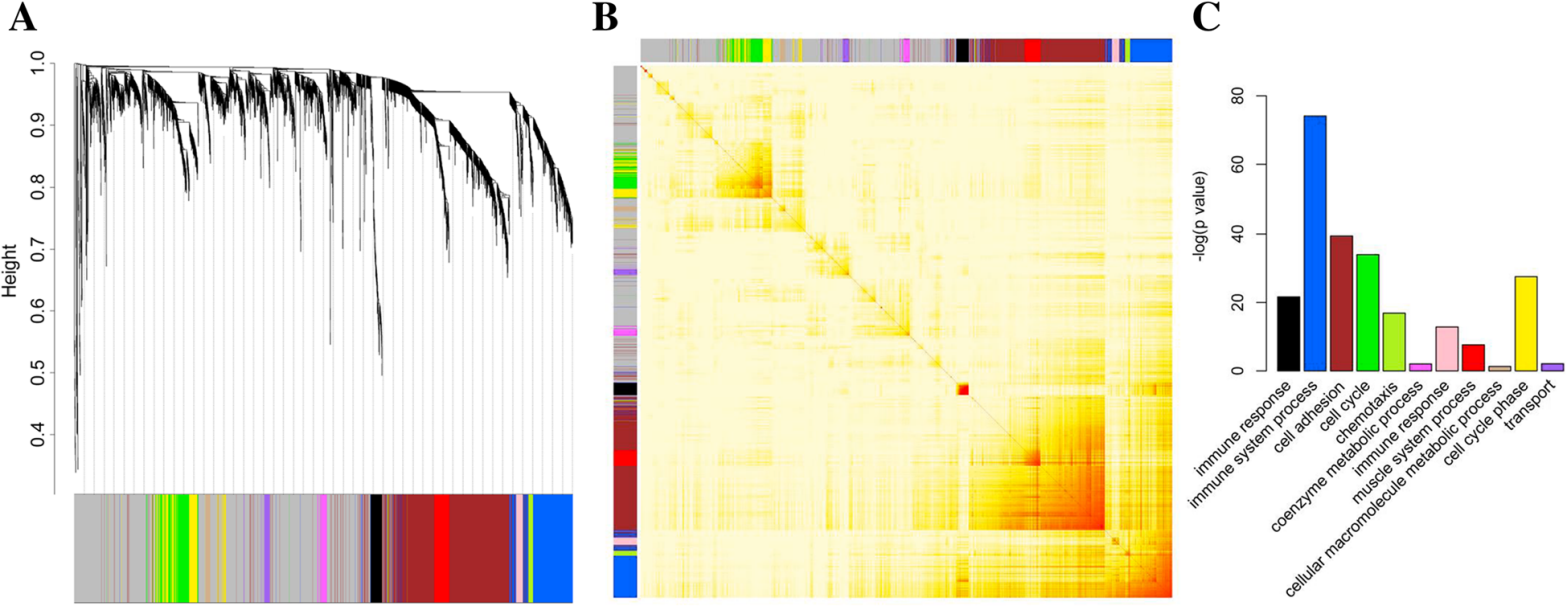
Identification of colon cancer specific modules using WGCNA, and GO enrichment analysis for these modules. **a** Clustering dendrogram of gene profilers from the testing dataset with 461 stage II or III colon cancer patients. Hierarchical cluster analysis dendrogram used to detect co-expression clusters. Each short vertical line corresponds to a gene, and the branches are expression modules of highly interconnected groups of genes with a colour to indicate its module assignment. A total of 11 modules ranging from 35 to 866 genes in size were identified. **b** Topological overlap matrix plot. Genes in the rows and columns are sorted by the clustering tree in (**a**). Clusters correspond to squares along the diagonal. **c** Bar plot presenting the top biological process enriched in each of the 11 modules. The original significance output from DAVID was transformed into “–log (*p*-value)” for plotting

WGCNA was performed to analyse 3600 gene expression profiles derived from 461 CC tumour tissues from the discovery dataset, to investigate the functional organisation of the CC transcriptome and to construct gene co-expression modules. A total of 11 gene modules were identified (Fig. 1a and b) from 35 to 866 genes (Table 1). Correlation or survival analysis was conducted to determine whether these modules are associated with tumour grade or RFS. The MEs, generated through principal component analysis, provide a general measure of the overall expression information in each module. Associations can then be determined on the basis of MEs. The module membership between each of the 3600 genes and the modules where these genes belong to (kMEs) was also calculated. The complete list of the network metrics (MEs and kMEs) and the module membership of each gene is shown in Additional file 2: Dataset 1.

**Table 1:** Association of expression modules with tumour stage and RFS in the discovery and validation datasets

Modules	Total gene count	Correlation with tumour stage		Association with RFS in the discovery dataset (*n* = 461)				Association with RFS in the validation dataset (*n* = 111)		
		R	*p*-value	HR	CI	*p*-value	FDR	HR	CI	*p*-value
Magenta	41	−0.07	1.46 × 10^−1^	0.84	0.63–1.13	2.52 × 10^−1^	3.90 × 10^−1^	0.55	0.30–1.00	5.13 × 10^−2^
Tan	35	0.05	3.21 × 10^−1^	0.82	0.60–1.12	2.11 × 10^−1^	3.86 × 10^−1^	1.38	0.73–2.59	3.20 × 10^−1^
Green	170	−0.14	2.64 × 10^−3^	0.59	0.42–0.81	1.37 × 10^−3^	7.52 × 10^−3^	0.51	0.25–1.05	6.67 × 10^−2^
Yellow	179	−0.05	2.62 × 10^−1^	0.59	0.43–0.81	9.60 × 10^−4^	7.52 × 10^−3^	0.70	0.37–1.34	2.82 × 10^−1^
Black	82	0.07	1.34 × 10^−1^	0.85	0.62–1.17	3.22 × 10^−1^	3.94 × 10^−1^	0.59	0.29–1.19	1.42 × 10^−1^
Purple	48	0.00	9.64 × 10^−1^	1.03	0.77–1.39	8.44 × 10^−1^	8.44 × 10^−1^	0.64	0.34–1.21	1.70 × 10^−1^
Brown	866	0.10	3.15 × 10^−2^	1.36	0.97–1.92	7.68 × 10^−2^	2.11 × 10^−1^	2.11	1.22–3.64	7.57 × 10^−3^
Red	108	0.11	2.12 × 10^−2^	1.33	0.94–1.87	1.03 × 10^−1^	2.27 × 10^−1^	1.12	0.58–2.14	7.36 × 10^−1^
Green-yellow	44	−0.03	5.29 × 10^−1^	0.84	0.61–1.16	2.84 × 10^−1^	3.90 × 10^−1^	1.27	0.70–2.29	4.35 × 10^−1^
Blue	369	0.04	4.32 × 10^−1^	0.93	0.66–1.32	6.99 × 10^−1^	7.69 × 10^−1^	1.56	0.80–3.01	1.89 × 10^−1^
Pink	52	0.03	5.91 × 10^−1^	0.65	0.47–0.91	1.08 × 10^−2^	3.97 × 10^−2^	1.35	0.72–2.53	3.50 × 10^−1^

*CI* 95% confidence interval, *RFS* recurrence-free survival. Hazard ratios (*HRs*), 95% CI, and *p*-values were calculated using Cox proportional hazard regression analysis

To determine the relationship among the 11 gene modules, we clustered their module eigengenes and associated these eigengenes with the GO annotation. Interestingly, the green and yellow modules contained the genes involved in cell cycle biological processes; among the modules, these two modules exhibited the closest connection in the cluster tree (Additional file 1: Figure S2).

### Gene modules are significantly correlated with RFS

**Fig. 2:**
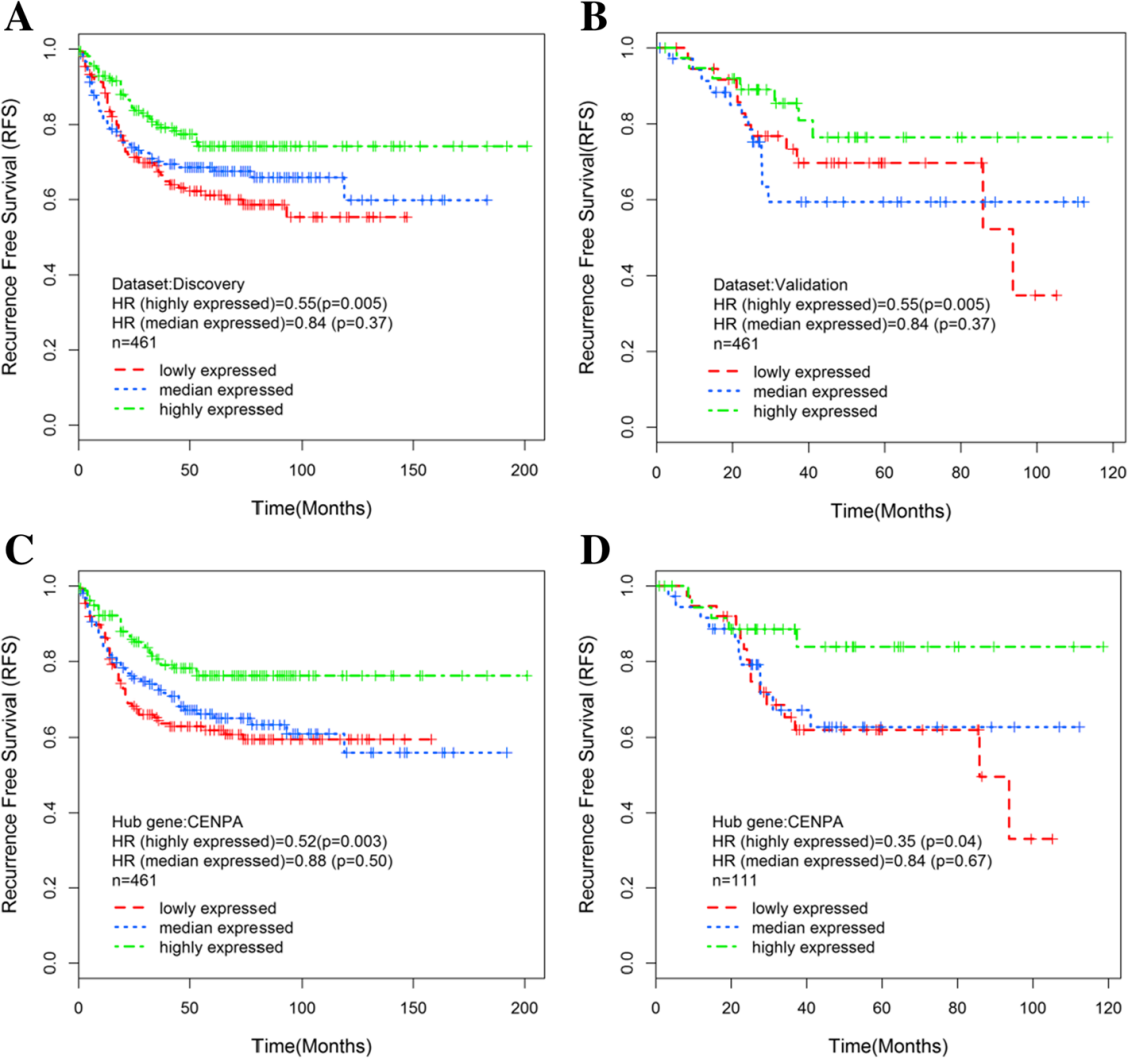
Associations between recurrence-free survival and the green module. The hub gene CENPA was observed in the testing and validating datasets. Kaplan–Meier survival plots for recurrence-free survival were shown (colon cancer patients were grouped by the tertile of ME). Increased expression (*green*) of the green module is associated with high recurrence-free survival in the testing (**a**) and validating (**b**) datasets. KM survival plot for CENPA suggests that its increased expression (*green*) indicates good prognosis (colon cancer patients were grouped by the tertile of gene expression level)(**c**, **d**)

The HRs and *p*-values of the dichotomised MEs were calculated through Cox regression to evaluate the relationship between RFS and co-expression modules (Table 1). The yellow, pink and green modules were significantly associated with RFS as a whole in the discovery dataset. However, only the association between the green module and RFS was confirmed in the validation dataset. The increased expression of genes in the green module indicated good prognosis (HR = 0.59, *p* = 1.37 × 10^−3^, FDR = 7.52 × 10^−3^ in the discovery dataset, HR = 0.51, *p* = 6.67 × 10^−2^ in the validation dataset, Fig. 2a, b). These findings suggested that a higher expression of the green module was associated with a low tumour grade (PCC = −0.14). After GO analysis was conducted, cell cycle-related biological processes were the most significantly overrepresented factor in the green co-expression module (Fig. 1c, Additional file 3: Dataset 2).

### Hub genes are associated with RFS

A total of 3600 genes were subjected to survival analysis to evaluate the effectiveness of WGCNA in the identification of novel hub genes that can be used as prognosis indicators. The HRs and the corresponding *p*-values of the genes included in the analysis of RFS are listed in Additional file 2: Dataset 1.

The increased expression of the green co-expression module containing 170 genes indicated an excellent RFS outcome (Table 1). In the single-gene survival analysis against RFS, 110 genes (Additional file 1: Table S2) that were significantly (*p* < 0.05) associated with good outcomes were identified in the green module. To the best of our knowledge, the relationship of the hub gene CENPA with RFS-related genes in CC has yet to be described. A high expression of the CENPA gene was associated with good RFS (HR = 0.62, *p* = 4.40 × 10^−4^ in the discovery dataset; HR = 0.53, *p* = 1.46 × 10^−2^ in the validation dataset, Table 2, Fig. c, d). To evaluate the robustness of the hub gene identification, we investigated the RFS-related modules and the corresponding hub genes with different initial gene selections (the most connected 3600 or 1800 genes) and cut height parameters (0.90 or 0.95) when identify gene modules through an average linkage hierarchical clustering method. CENPA could be identified under these conditions (Additional file 1: Table S2).

**Table 2:** Relationships between hub genes with RFS in the discovery and validation datasets

Hub gene	k.in rank	Association with RFS in the discovery dataset (*n* = 461)				Association with RFS in the validation dataset (*n* = 111)		
		HR	95% CI	*p*-value	FDR *p*-value	HR	*p*-value	CI
CDCA5	1	0.70	0.55–0.89	4.32 × 10^−3^	5.51 × 10^−3^	0.69	1.66 × 10^−1^	0.40–1.17
NCAPH	2	0.67	0.52–0.86	1.58 × 10^−3^	3.68 × 10^−3^	0.64	1.01 × 10^−1^	0.37–.09
FEN1	4	0.68	0.52–0.88	3.43 × 10^−3^	5.51 × 10^−3^	0.70	1.97 × 10^−1^	0.41–1.21
MCM2	5	0.70	0.55–0.90	4.72 × 10^−3^	5.51 × 10^−3^	0.79	3.98 × 10^−1^	0.46–1.36
MCM10	6	0.69	0.54–0.90	5.95 × 10^−3^	5.95 × 10^−3^	0.73	2.72 × 10^−1^	0.41–1.28
CENPA	7	0.62	0.48–0.81	4.40 × 10^−4^	2.80 × 10^−3^	0.53	1.46 × 10^−2^	0.31–0.88
ZWINT	9	0.64	0.50–0.83	8.00 × 10^−4^	2.80 × 10^−3^	0.63	8.60 × 10^−2^	0.37–1.07

k.in rank: gene rank based on k.in; False discovery rate (*FDR*); Recurrence-free survival (*RFS*); Hazard ratios (*HRs*), 95% confidence intervals (*CI*), and *p*-values were calculated using Cox proportional hazard regression analysis treating the gene expression level as a continuous variable

### Gene modules are significantly associated with CC subtype-specific survival

In addition to the survival analysis of CC as a whole, the investigation of the association between gene modules and molecular subtypes was conducted. Survival analysis revealed that the yellow, red and tan modules were associated with type 3 (Table 3). The increased expression of the yellow (HR = 0.38, *p* = 4.46 × 10^−3^) and tan (HR = 0.50, *p* = 3.39 × 10^−2^) co-expression modules containing 179 and 35 genes were correlated with good RFS outcomes. While the increased expression of the red co-expression module containing 108 genes was correlated with poor RFS outcomes for the type 3 (HR = 1.74, *p* = 3.02 × 10^−2^).

**Table 3:** Relationship between expression modules with RFS within colon cancer molecular subtypes in the discovery dataset

Modules	Total gene count	Type 3 (*n* = 108)			Type 4 (*n* = 151)		
		HR	95% CI	*p*-value	HR	95% CI	*p*-value
Magenta	41	0.99	0.52–1.88	9.78 × 10^−1^	0.81	0.44–1.46	4.77 × 10^−1^
Tan	35	0.50	0.26–0.95	3.39 × 10^−2^	0.90	0.53–1.56	7.17 × 10^−1^
Green	170	0.74	0.37–1.46	3.83 × 10^−1^	0.87	0.47–1.60	6.53 × 10^−1^
Yellow	179	0.38	0.20–0.74	4.46 × 10^−3^	0.89	0.50–1.58	6.87 × 10^−1^
Black	82	0.81	0.43–1.54	5.27 × 10^−1^	0.80	0.45–1.41	4.44 × 10^−1^
Purple	48	1.46	0.76–2.77	2.54 × 10^−1^	1.05	0.63–1.76	8.44 × 10^−1^
Brown	866	1.61	0.92–2.83	9.50 × 10^−2^	1.38	0.72–2.67	3.34 × 10^−1^
Red	108	1.74	1.05–2.88	3.02 × 10^−2^	1.15	0.61–2.18	6.63 × 10^−1^
Green-yellow	44	1.26	0.59–2.67	5.52 × 10^−1^	0.95	0.49–1.83	8.73 × 10^−1^
Blue	369	1.02	0.45–2.34	9.58 × 10^−1^	1.05	0.53–2.09	8.92 × 10^−1^
Pink	52	0.51	0.23–1.10	8.57 × 10^−2^	0.63	0.34–1.20	1.59 × 10^−1^

Type 3 definition: MSI-low, CIMP-negative, negative for BRAF mutation, positive for KRAS mutation; Type 4 definition: MSI-low, CIMP-negative, negative for mutations in BRAF and KRAS. False Discovery Rate (*FDR*); Recurrence-free survival (*RFS*); Hazard ratios (*HRs*), 95% confidence intervals (*CI*), and *p*-values were calculated using Cox proportional hazard regression analysis, treating the MEs as continuous variables

The HRs and the corresponding *p*-values of the single gene survival for the type 3 and type 4 subtypes (Addtional file 2: Dataset 1) were also calculated. The yellow module genes in the type 3 group were analysed, and the results revealed three hub gene prognosis indicators, including cyclin-dependent kinase 1 (CDK1; HR = 0.52, *p* = 3.42 × 10^−3^), kinesin family member 11 (KIF11; HR = 0.57, *p* = 5.76 × 10^−3^) and RAD51 associated protein 1 (RAD51AP1; HR = 0.52, *p* = 1.42 × 10^−3^). Meanwhile, we analysis the tan module genes in the type 3 group, and found hub gene OTU domain containing 6B (OTUD6B; HR = 0.56, *p* = 6.24 × 10^−3^). The increased expression of these genes suggested good prognosis in the type 3 subtype. There was no gene met the hub gene defination criterion in module tan for type 3 subtype.

## Discussion

We applied a systems biology approach, namely, WGCNA, to analyse one mRNA expression dataset comprising 461 CC patients to identify the networks and genes associated with clinical variables and prognosis indicators. We then confirmed our findings by using an independent validation dataset. WGCNA can be applied to determine complex biological mechanisms responsible for the target phenotypes; this method is effective because the algorithm aims to clarify the relationships between genes above noise and maintain consistency among all of the samples. The unsupervised hierarchical clustering method selected by WGCNA avoids potential biases and subjective decisions attributed to the selection of the candidate genes previously reported as associated with CC or to the early distinction of control samples for supervised methods.

In our study, 11 distinct gene modules from 3600 genes that satisfied our pre-filtering standard for the co-expression analysis were identified. The increased expression of the green module containing 170 genes mostly related to the cell cycle was associated with low tumour grade and correlated with positive RFS outcome. The association relationship reached statistical significance in the discovery dataset (*p* = 1.37 × 10^−3^, FDR = 7.52 × 10^−3^), and marginal significance (*p* = 6.67 × 10^−2^) in the validation dataset. This marginal significance may account for the small sample size (*n* = 111) and lack of statistical power. Since we can make conclusions depend on the effect size and its precision rather than just the *p*-value [26]. The result of HR and 95% CI of module green in the validation dataset suggested that the association relationship between module green and RFS of CC patients was clinically significance. After conducting the single gene survival analysis of each member of the green module, we found that approximately 65% of the genes were significantly related to RFS (*p* < 0.05), and all of the genes yielded HR < 1. Furthermore, the hub gene CENPA was identified as a potential novel marker. CENPA, a protein-coding gene, is the histone-H3-like variant essential for centromere functioning and structure. This gene is implicated in cell cycle and mitotic pathways; the GO annotations of this gene include protein heterodimerisation activity and chromatin binding. CENPA is also a potential prognostic biomarker of breast cancer, and the increased expression of this gene is associated with the poor survival of breast cancer patients [27, 28]. Among various core markers in neoplasic intratubullar germ cells, such as CD9, CENPA and PODXL, CENPA is overexpressed, and this finding suggests that this gene may be a potential biological marker of human diseases [29]. Tomonaga et al. demonstrated that CENPA is overexpressed at a transcriptional level in all 11 primary human CC tissues [30]. Furthermore, the immunostaining with anti-CENPA antibodies revealed that the CENPA signals in tumour cells increase; therefore, the overexpression of CENPA may be critical in aneuploidy in colorectal cancers [30]. However, the role of CENPA in CC should be further validated.

The yellow module containing 179 genes involved in the cell cycle was correlated with RFS in the type 3 subtypes, including MSI-low, CIMP-negative, negative for BRAF mutation and positive for KRAS mutation. We also identified CDK1 as a marker. The specific activity of CDK1 is a promising biomarker of the metastasis risk in stage II CC [31]. The type 3 subgroup is the only subgroup with KRAS mutation. KRAS, a proto-oncogene, encodes a small 21 kD guanosine triphosphate/guanosine diphosphate binding protein that modulates cellular proliferation and differentiation [32]. Approximately 97% of KRAS mutations are caused by seven different DNA base-pair substitutions in codons 12 and 13 of exon 2; as a result, an amino acid substitution in the protein occurs [33]. Therefore, KRAS may affect cell cycle processes. In a recent study, CDK1 is reported as a synthetic lethality target for KRAS mutation in colon cancer [34]. Our study highlisht RAD51AP1 as a prognostic marker and therapeutic target. It has been reported that Overexpression of RAD51 is a negative prognostic marker for colorectal adenocarcinoma [35]. However, the roles of the hub gene KIF11 in module yellow and OTUD6B in module tan in CC have yet to be determined.

As a retrospective study, the current study is characterised by several limitations. Firstly, relevant information, such as the accurate definition of RFS, the MSI/CIMP status and the molecular subtypes of the validation dataset, was unavailable. As such, the associations between modules and RFS in each subtype could not be validated. Secondly, although the direction of the association between the green module and RFS in the validation dataset was similar to that in the discovery dataset, the *p*-value was marginally significant. Thus, the significance and robustness of the network and hub genes should be confirmed in prospective clinical trials, ideally with large prospective patient cohorts.

## Conclusions

In summary, 11 gene co-expression modules were identified from an mRNA microarray-based study through WGCNA. We associated these gene modules to tumour grade and RFS. We also evaluated the prognostic ability of single genes through Cox-regression analysis. Moreover, a co-expression module indicative of patients’ RFS for a particular molecular marker-based subtype, such as KRAS mutation group, was identified. Indeed, WGCNA is an effective technique that can be applied to investigate the underlying biological mechanisms and identify the genes indicative of patient outcome. The practical utility of this approach is exemplified through the identification of novel prognostic makers, such as CENPA. Our investigation could contribute to personalised therapies. Nevertheless, multicenter randomised controlled clinical trials and in vivo/in vitro experiments should be performed to evaluate the possible applications of molecular signatures to predict survival and to functionally characterise the hub genes for clinical applications.

## Additional files

Additional file 1: Table S1. Summary of colon cancer microarray datasets used in the study. **Table S2.** Identified significant module when use different number of genes and cut height parameter. **Figure S1.** Criteria for choosing the beta parameter. **Figure S2.** Clustering plot of module eigengenes. (DOC 81 kb)

Additional file 2: Dataset 1. WGCNA and survival analysis for the 3600 genes contained in the 11 co-expression modules. The kME and k.in with the parent module and the survival calculation for RFS and molecular subtypes (type 3 and type 4) are presented. (XLS 3080 kb)

Additional file 3: Dataset 2. GO biological process enrichment analysis for the 11 modules. (XLS 406 kb)

## Abbreviations

CC: Colon cancer
CI: Confidence interval
CIMP: CpG island methylator phenotype
FDR: False discovery rate
GEP: Gene expression profile
GO: Gene ontology
HR: Hazard ratio
ME: Module eigengene
MSI: Microsatellite instability
RFS: Recurrence-free survival
TNM: Tumour, node and metastasis
TOM: Topological overlap matrix
WGCNA: Weighted gene co-expression network analysis
