# Supplementary material for: Associating transcriptional modules with colon cancer survival through weighted gene co-expression network analysis
Source: BMC Genomics. 2017 May 9;18:361. doi: 10.1186/s12864-017-3761-z (PMC5424422; doi:10.1186/s12864-017-3761-z)
Supplement: Supplementary file 1 — Summary of colon cancer microarray datasets used in the study. Table S2. Identified significant module when use different number of genes and cut height parameter. Figure S1. Criteria for choosing the beta parameter. Figure S2. Clustering plot of module eigengenes. (DOC 81 kb) [file 12864_2017_3761_MOESM1_ESM.doc]

**Associating transcriptional modules with colon cancer survival through weighted gene co-expression network analysis**

Rong Liu ^1*^

1. Department of Clinical Pharmacology, Xiangya Hospital, Central South University, Changsha 410008; P. R. China; Institute of Clinical Pharmacology, Central South University; Hunan Key Laboratory of Pharmacogenetics, Changsha 410078; P. R. China.

#Correspondence: Dr. Rong Liu.

Department of Clinical Pharmacology, Xiangya Hospital, Central South University, Changsha 410008; P. R. China; Institute of Clinical Pharmacology, Central South University; Hunan Key Laboratory of Pharmacogenetics, Changsha 410078; P. R. China.

E-mail: [yezi19870127@163.com](mailto:yezi19870127@163.com)

**Additional file 1**

**Table S1** Summary of colon cancer microarray datasets used in our study.

| **GEO**  **Accession** | **Ref.** | **Sample**  **#** | **Sample used*** | **Tumour grade** | | **Age**  **(Years)** | **Survival (months)** | **Information** | | |
| --- | --- | --- | --- | --- | --- | --- | --- | --- | --- | --- |
|  |  |  |  | **2** | **3** |  |  | **BRAF** | **KRAS** | **TP53** |
| **GSE39582** | Laetitia Marisa *et al*. 2013 | 566 | 461 | 260 | 201 | 68 ± 13 | 53.08 ± 40.36 | 🗸 | 🗸 | 🗸 |
| **GSE17536** | Smith JJ *et al*. 2010 | 177 | 111 | 55 | 56 | 65 ± 14 | 43.05 ± 27.89 | 🗴 | 🗴 | 🗴 |

*We just used the patients in stage II-III with survival data.

**Table S2** Identified significant module when use different number of genes and cut height parameter.

| **Number of Genes** | **Cut height** | **Module** | | | | | | | **Biological process** | **Hub genes in this module** |
| --- | --- | --- | --- | --- | --- | --- | --- | --- | --- | --- |
|  |  | **colour** | **N** | **Testing dataset** | | | **Validating dataset** | |  |  |
|  |  |  |  | **HR** | **P value** | **FDR** | **HR** | **P value** |  |  |
| **3600** | 0.90 | Black | 136 | 0.57 | 7.54×10^–4^ | 4.52×10^–3^ | 0.56 | 9.27×10^–2^ | Cell cycle | CCNA2, **CENPA**, MCM10, NCAPG, NCAPH, ZWINT |
|  | 0.95 | Green | 170 | 0.59 | 1.37×10^–3^ | 7.52×10^–3^ | 0.51 | 6.67×10^–2^ | Cell cycle | CDCA5, NCAPH, FEN1, MCM2, MCM10, **CENPA**, ZWINT |
| **1800** | 0.90 | Green | 119 | 0.57 | 7.72×10^–4^ | 3.86×10^–3^ | 0.57 | 1.05×10^–1^ | Cell cycle | CCNA2, **CENPA**, MCM10, NCAPG, NCAPH, ZWINT |
|  | 0.95 | Red | 97 | 0.58 | 1.05×10^–3^ | 4.18×10^–3^ | 0.52 | 7.08×10^–2^ | Cell cycle | CDCA5, **CENPA**, FEN1, MCM10, MCM2, NCAPH, ZWINT |


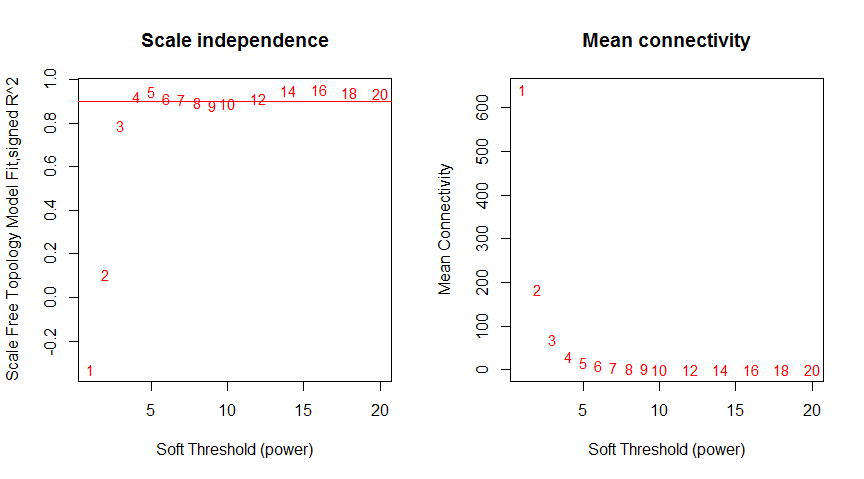


**Figure S1**: **Criteria for choosing the beta parameter**. For different soft thresholds (bottom row, beta), the left plot visualize the scale free topology fitting index, while the right plot visualize the mean connectivity. There is a trade-off between a high scale-free topology fit (R^2^) and a high mean number of connections.


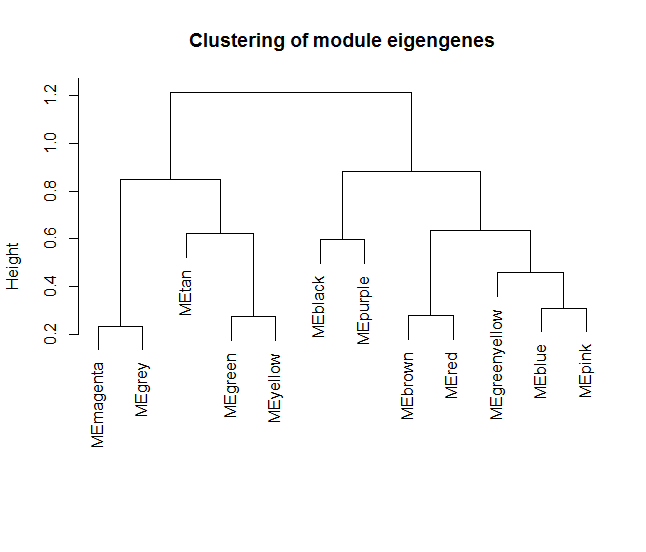
**Figure S2. Clustering plot of module eigengenes**.
